# Hordenine Protects Against Lipopolysaccharide-Induced Acute Lung Injury by Inhibiting Inflammation

**DOI:** 10.3389/fphar.2021.712232

**Published:** 2021-09-01

**Authors:** Xiyue Zhang, Li Du, Jinrong Zhang, Chunyan Li, Jie Zhang, Xuejiao Lv

**Affiliations:** ^1^ Department of Respiratory and Critical Care Medicine, The Second Hospital of Jilin University, Changchun, China; ^2^ Department of Pathogeny Biology, College of Basic Medical Sciences, Jilin University, Changchun, China

**Keywords:** acute lung injury, hordenine, lipopolysaccharide, protein kinase B/nuclear factor-κB, mitogen-activated protein kinase, AutoDock

## Abstract

Acute lung injury (ALI) is a respiratory disease that leads to death in severe cases. Hordenine (Hor), a barley-derived natural product, has various biological activities, including anti-inflammatory, and anti-oxidation activities. We investigated the effect of Hor on lipopolysaccharide-induced ALI and its potential mechanism. The anti-inflammatory effects of Hor were detected using *in vivo* and *in vitro* models by enzyme-linked immunosorbent assay, real-time polymerase chain reaction, western blotting, and molecular docking simulations. Hor inhibited increases in the levels of inflammatory factors both *in vivo* and *in vitro*, and its anti-inflammatory effect inhibited activation of protein kinase B, nuclear factor-κB, and mitogen-activated protein kinase signaling. Hor alleviated lipopolysaccharide-induced ALI by inhibiting inflammatory cytokine increases *in vivo* and *in vitro* and shows potential for preventing inflammatory disease.

## Introduction

Acute lung injury (ALI) and acute respiratory distress syndrome are respiratory diseases in which the lung tissue is damaged, accompanied by a strong inflammatory response (Butt et al., 2016; Komiya et al., 2017). ALI may be caused by many different factors, including chemical stimulation, mechanical injury, and virus infection (Dushianthan et al., 2011; Butt et al., 2016). Increased capillary permeability from lung tissue edema, inflammatory cell infiltration during parenchymal organ necrosis, lung barrier destruction, microbial infection, and dyspnea caused by hypoxemia are the main characteristics of ALI (Nova et al., 2019; Mowery et al., 2020). This disease has a high mortality rate and there is no effective treatment in clinical practice; thus, effective alleviation of ALI is an urgent problem (Vlaar and Juffermans, 2013; Komiya et al., 2017).

Inflammation plays an important role in ALI (Henes et al., 2009; Vlaar and Juffermans, 2013). Macrophages in lung tissues are important in inflammation, and macrophage activation leads to the production of inflammatory mediators such as myeloperoxidase (MPO), interleukin (IL)-6, IL-1β, tumor necrosis factor (TNF)-α, inducible nitric oxide synthase (iNOS), and cyclooxygenase-2 (COX-2) (Lv et al., 2017; Hou et al., 2018). These inflammatory mediators stimulate and damage lung tissue cells, resulting in lung tissue cell necrosis, and impaired function (Zhong et al., 2013; Dias-Freitas et al., 2016; Lv et al., 2017). Therefore, effective control of inflammatory mediator levels is a potential therapeutic approach for alleviating ALI (Lv et al., 2017; Huang et al., 2019). Nuclear factor (NF)-κB and mitogen-activated protein kinase (MAPK) are two classical signaling pathways involved in inflammation; activation of NF-κB and MAPK aggravates the secretion of inflammatory mediators and then expands the promoting effect of the inflammatory response on ALI (Zhong et al., 2013; Zhang et al., 2015). Currently, NF-κB and MAPK are potential therapeutic targets for mitigating ALI (Zhong et al., 2013; Jing et al., 2015). Lipopolysaccharide (LPS) is a bacterial endotoxin; because of its strong immunogenicity, it causes the body’s immune cells to produce a strong inflammatory response (Kan et al., 2019). LPS-induced lung tissue inflammation in mice has been widely used as an ALI model (Zhong et al., 2013; Lv et al., 2017), including for screening of candidate clinical drugs for ALI disease (Seehase et al., 2012; Hou et al., 2018; Zhang et al., 2018).

In recent years, the treatment of ALI with natural products has become a new research direction (Zhong et al., 2013; He et al., 2021b). Hordenine (Hor), with the chemical name 4-(2-dimethylaminoethyl), is a natural phenolic phytochemical compound extracted from germinated barley and is found in plants such as cactus and bitter orange (Kim et al., 2013); it shows potential as a drug candidate for the treatment of inflammatory diseases (Zhou et al., 2019). Hor has been found to have anti-inflammatory effects in diabetic nephropathy (Su et al., 2018) and to be a quorum-sensing inhibitor against *Pseudomonas aeruginosa* and *Serratia marcescens* (Zhou et al., 2018; Zhou et al., 2019). However, whether Hor alleviates LPS-induced ALI is unknown. Therefore, we used an LPS-induced ALI disease model to explore the effects of Hor on LPS-induced ALI and its potential mechanisms. This study provides a theoretical basis and guidance for alleviating ALI.

## Materials and Methods

### Reagents

Hor (high-performance liquid chromatography-grade ≥95%) was purchased from Shanghai Yuanye Bio-Technology Co., Ltd. (Shanghai, China). Dimethyl sulfoxide and LPS were purchased from Sigma Aldrich (St. Louis, MO, United States). Phosphate-buffered saline and 0.05% pancreatic enzyme were purchased from Solarbio Technology (Beijing, China). Penicillin and streptomycin, fetal bovine serum, and Dulbecco’s modified Eagle’s medium (DMEM) were purchased from Hyclone Laboratories (Logan, UT, United States). Primary antibodies [COX-2, iNOS, protein kinase B (AKT), p-AKT, NF-κB-p65, NF-κB-p-p65, inhibitor of NF-κB (IκB), p-IκB, p38, p-p38, c-Jun N-terminal kinase (JNK)1/2, p-JNK1/2, extracellular signal-regulated kinase (ERK)1/2, and p-ERK1/2] were purchased from Cell Signaling Technology (Danvers, MA, United States), and primary antibodies against β-actin and goat anti-mouse or goat anti-rabbit secondary antibodies were obtained from Santa Cruz Biotechnology (Dallas, TX, United States).

### Drug Management

Hor powder was dissolved in dimethyl sulfoxide at a concentration of 1 g/ml and stored until use. Considering drug toxicity and the pre-experimental screening of the dose, 10 and 15 mg/kg were found to be safe for mice. So Hor at doses of 10 and 15 mg/kg was used for animal experiments, whereas 50 and 75 μg/ml Hor were used for cell experiments.

### Animals

Male BALB/c mice (*n* = 75) aged 6–8 weeks were purchased from HFK Bioscience (Beijing, China). The mice had free access to food and water, and were subjected to a 12 h light/dark cycle. Three mice were placed in each cage. The mice were divided into five groups: no treatment group (NT), Hor, LPS, LPS + 10 mg/kg Hor, and LPS + 15 mg/kg Hor. During the experiment, all operations were strictly performed in accordance with the guidelines of the Institutional Animal Care and Use Committee of Jilin University (Changchun, China) (Permit Number: SY202106009).

### Animal Treatment

The ALI mouse model was established by intranasal instillation of LPS (1.25 mg/kg soluble in 20 µL phosphate-buffered saline). Mice in the NT and LPS groups were pre-treated by an intraperitoneal injection of Hor for 1 h; the same volume of solvent was injected in the NT group. Before constructing the model, the anesthetic sodium pentobarbital (45 mg/kg) was prepared, and each mouse was injected with 100 μL of the anesthetic. After the mice were anesthetized, they were fixed in the supine position; LPS was added to one side of the nostril during the deep and fast breathing period, and then the mice were gently rotated to evenly distribute the LPS to the lungs. Lung tissue was collected at 24 h after LPS infusion for subsequent experiments.

### Lung Tissue Pathological Examination

Fresh lung tissues of mice were immersed in 4% formaldehyde solution for 48 h and then dehydrated with alcohol. After soaking in paraffin, the tissues were sliced into 5-μm-thick sections and stained with hematoxylin and eosin (H and E).

### Injury Score

Lung tissue injury was assessed by light microscopy and evaluated according to the size of the alveolar stromal space, inflammatory cell infiltration, hemorrhage, and edema. A score of 0 was given for no injury, one for mild injury, two for moderate injury, three for severe injury, and four for extreme injury.

### Ratio of Lung Wet-Dry Weight

The fresh lung tissue was weighed and recorded. The samples were weighed and recorded after 48 h in an incubator at 65°C. Finally, the W/D weight ratio was calculated.

### MPO Activity Measurement

The mouse lung tissue was collected in a 2 ml centrifuge tube and weighed. Lung tissue (1 g) was added to 4 ml 0.5% cetyltrimethylammonium chloride solution, and then placed into a grinding instrument for grinding. The supernatant was collected after centrifugation at 12,000 × *g* for 10 min. A total of 75 µL supernatant and 75 µL working solution were successively added to a 96-well plate and termination solution was added after incubation for 20 min. The absorbance was measured at 450 nm using a microplate analyzer (BioTek, Winooski, VT, United States).

### Cell Culture and Treatment

RAW264.7 macrophage cells were purchased from China Cell Line Bank (Beijing, China) and cultured in DMEM containing 10% fetal bovine serum with 1% penicillin and streptomycin. The cells were cultured in a constant-temperature incubator at 37°C and 5% CO_2_. When the cells reached 80% confluence, they were treated with 0.05% trypsin and sub-cultured at a ratio of 1:3. The media were replaced with serum-free medium at 3 h before treatment to reduce mitosis. Before LPS stimulation (1 μg/ml), the cells were pre-treated with different concentrations of Hor for 1 h.

### Cell Viability Assay

When the fusion density reached 80%, the cells were digested for the experiment. The Cell Counting Kit-8 (CCK8) assay was used to determine the effect of Hor on the viability of RAW264.7 cells. Different concentrations of Hor (12.5–100 μg/ml) were added to 96-well plates. After 24 h, the medium was discarded and 10 μL of CCK8 solution was added to each well. After 2 h, the absorbance was measured at 450 nm using a microplate analyzer.

### Reverse Transcription-Quantitative Polymerase Chain Reaction

To extract RNA, 1 ml of TRIzol solution was added to the lung tissue or cell culture dishes after discarding the DMEM, chloroform was added after 10 min, and then the supernatant was centrifuged at 12,000 × *g* to obtain 300 μL supernatant. Isopropanol was added to precipitate the RNA in the supernatant. Next, 75% diethyl pyrocarbonate alcohol was added to precipitate the total RNA; RNA precipitates were added to diethyl pyrocarbonate water before concentration measurements, and the samples were stored at −80°C. RNA was reverse-transcribed into cDNA using two reverse transcription steps as described previously (Kan et al., 2019) using an RT-PCR Kit from Takara Biomedical Technology Co., Ltd. (Shiga, Japan). The primer sequences are shown in Table 1, as previously reported (Hou et al., 2018; He et al., 2021a).

**TABLE 1:** Primer sequences for reverse transcription-quantitative polymerase chain reaction.

Primer name	Sequence (5′ to 3′)
IL-6-F	CCA​GAA​ACC​GCT​ATG​AAG​TTC​C
IL-6-R	GTT​GGG​AGT​GGT​ATC​CTC​TGT​GA
TNF-α-F	ACG​GCA​TGG​ATC​TCA​AAG​AC
TNF-α-R	GTG​GGT​GAG​GAG​CAC​GTA​GT
IL-1β-F	GTT​CCC​ATT​AGA​CAA​CTG​CAC​TAC​AG
IL-1β-R	GTC​GTT​GCT​TGG​TTC​TCC​TTG​TA
iNOS-F	GAA​CTG​TAG​CAC​AGC​ACA​GGA​AAT
iNOS-R	CGT​ACC​GGA​TGA​GCT​GTG​AAT
COX-2-F	CAG​TTT​ATG​TTG​TCT​GTC​CAG​AGT​TTC
COX-2-R	CCA​GCA​CTT​CAC​CCA​TCA​GTT
Arg-1-F	GTG​AAG​AAC​CCA​CGG​TCT​GT
Arg-1-R	GCC​AGA​GAT​GCT​TCC​AAC​TG
Ym-1-F	CAG​GGT​AAT​GAG​TGG​GTT​GG
Ym-1-R	CAC​GGC​ACC​TCC​TAA​ATT​GT
CD206-F	CTT​CGG​GCC​TTT​GGA​ATA​AT
CD206-R	TAG​AAG​AGC​CCT​TGG​GTT​GA
β-actin-F	GTC​AGG​TCA​TCA​CTA​TCG​GCA​AT
β-actin-R	AGA​GGT​CTT​TAC​GGA​TGT​CAA​CGT

### Inflammatory Cytokine Assay

One milliliter of 0.9% normal saline was injected into the lungs of mice through the bronchial tubes, and 800 μL bronchoalveolar lavage fluid (BALF) was recovered in a 1.5 ml centrifuge tube. The supernatant was collected after centrifugation at 10,000 × *g* for 5 min. RAW264.7 cells were pre-treated with Hor (50 and 75 μg/ml) for 1 h and stimulated with LPS (1 μg/ml) for 12 h, after which the medium was collected and centrifuged at 10,000 × *g* for 5 min to collect the supernatant. TNF-α, IL-6, and IL-1β protein levels were determined using an enzyme-linked immunosorbent assay kit (BioLegend, San Diego, CA, United States) according to the manufacturer’s instructions.

### Western Blotting

Radioimmunoprecipitation assay buffer (Roche Diagnostics, Basel, Switzerland) was added to lyse the cells. The supernatant was centrifuged at 12,000 × *g* for 10 min to obtain the total protein solution. A bicinchoninic acid protein assay kit (Thermo Fisher Scientific, Waltham, MA, United States) was used to determine the protein concentration; each 15 μL sample contained 40 μg of protein. After electrophoresis, the proteins in the gel were transferred onto polyvinylidene fluoride membranes (Millipore, Billerica, MA, United States), which were incubated with primary antibodies (1:1,000) overnight at 4°C. Secondary antibodies (1:2,000) were added to the membranes and incubated for 1 h the next day. Finally, an enhanced chemiluminescence kit (Applygen Inst. Biotech, Beijing, China) was used to detect the protein bands.

### Molecular Modeling

Three-dimensional structure information for AKT protein was obtained from the Protein Data Bank. Hor information was obtained from the PubChem database and downloaded in SDF format. AutoDock tools were used to simulate the calculation of amino acid residues with hydrogen bonds between AKT and Hor. PyMOL was used to visualize the results.

### Immunofluorescence Measurements

A clean sterile cover glass was evenly spread on a 24-well plate, and the cells were evenly inoculated onto the slide. Hor (75 μg/ml) was added to the medium; after 1 h, LPS (1 μg/ml) was added to the medium. After 12 h, the cells were placed on an experimental bench for immunofluorescence analysis.

The cells were fixed in 4% formaldehyde and then 0.1% Triton X-100 was added to the cell surface. The cells were blocked with 5% donkey serum for 2 h. The primary antibody (p65, 1:200) was added to the glass coverslip, and the cover glass was placed in a wet box at 4°C overnight. The next day, the fluorescent secondary antibody (1:2,000) was dripped onto the surface of the cover glass slide and the slide was incubated in the dark. After adding 4′,6-diamidino-2-phenylindole, p65 transfer into the nucleus was observed under a fluorescence microscope.

### Data and Statistical Analysis

One-way analysis of variance was used to analyze the data with GraphPad Prism 8. The results are expressed as the mean ± standard deviation (SD). Adobe Photoshop CC 2017 was used for image processing. AutoDock tools-1.5.6 and PyMOL were used for molecular docking simulation. ImageJ software (NIH, Bethesda, MD, United States) was used for quantitative analysis of protein bands.

## Results

### Hor Alleviated Lung Pathological Damage in LPS-Induced ALI

**FIGURE 1:**
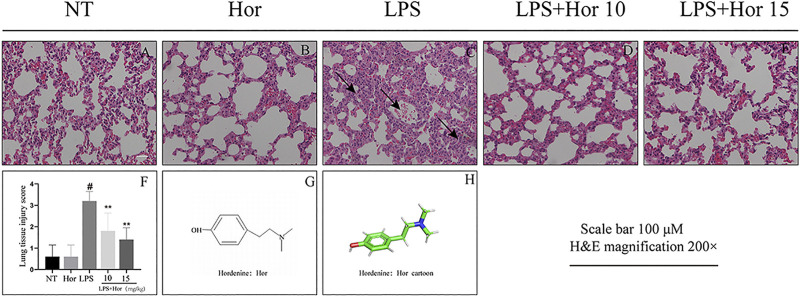
Hordenine (Hor) alleviates lung pathological damage in LPS-induced acute lung injury (ALI). After intraperitoneal injection of Hor (5 and 10 mg/kg) at 1 h before modeling, an acute lung injury model was established using nasal drops. Lung tissue was collected at 24 h after LPS stimulation. Tissue sections were stained with hematoxylin and eosin (H and E). **(A–E)** H and E staining results of the lung tissue in mice (arrows represent the lesion area). **(F)** Lung histopathological injury scores. **(G)** Hor molecular structure. **(H)** Three-dimensional model of the Hor molecular structure. Results are shown as means ± SD (*n* = 3). #*p* < 0.01 vs. no treatment (NT) group; ^∗∗^ *p* < 0.01 vs. LPS group. Hor: hordenine; LPS: lipopolysaccharide.

To explore whether Hor alleviated ALI, damage to the lung tissue was evaluated by H and E staining. Compared to the NT group, LPS significantly induced lung inflammatory infiltration, alveolar damage, and alveolar interstitial thickening. However, after Hor pre-treatment, these pathological symptoms were relieved; with increasing Hor concentrations, the alleviation of ALI was more significant (Figure 1).

### Effect of Hor on the W/D Weight Ratio of Lung Tissue in LPS-Induced ALI

**FIGURE 2:**
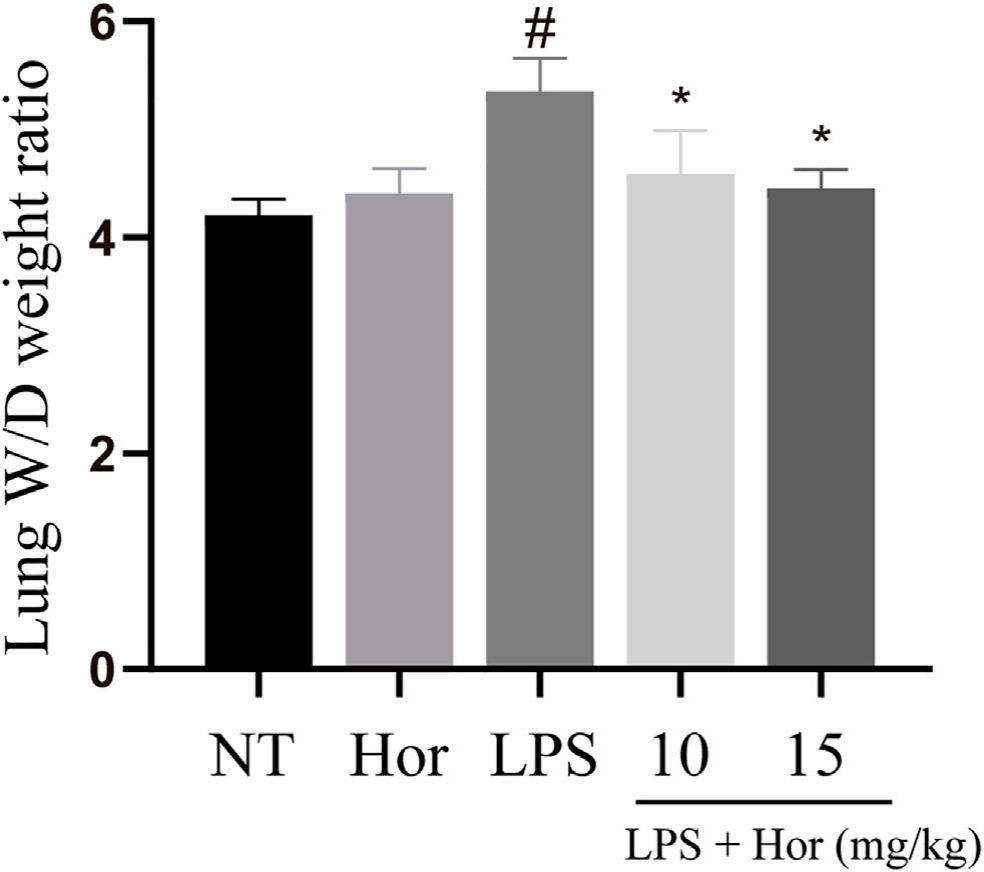
Hordenine alleviates the wet-dry ratio weight of lung tissue in LPS-induced acute lung injury. Fresh lung tissues were collected at 24 h after the LPS challenge. Results are shown as means ± SD (*n* = 3). #*p* < 0.01 vs. no treatment (NT) group; ^∗^ *p <* 0.05 vs. LPS group. Hor: hordenine; LPS: lipopolysaccharide; W/D: wet-dry.

LPS significantly induced pulmonary edema compared to the NT group. However, Hor pre-treatment effectively alleviated LPS-induced pulmonary edema at both the low and high concentrations (Figure 2).

### Hor Reduced the Expression and Secretion of Inflammatory Cytokines in the Lung Tissue and BALF in an LPS-Induced Mouse Model

**FIGURE 3:**
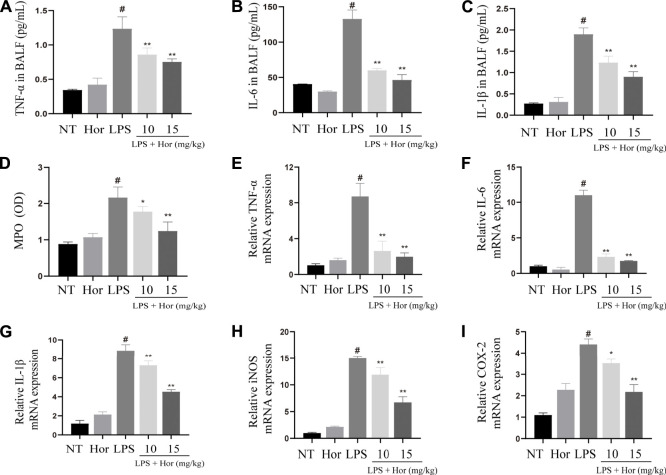
Hordenine inhibits the secretion and activation of inflammatory cytokines in LPS-induced acute lung injury. BALF and lung tissues were collected at 24 h after LPS infusion. **(A–C)** Levels of IL-6, IL-1β, and TNF-α in the BALF of mice. **(D)** MPO activity in lung tissues. **(E–I)** mRNA expression of inflammatory cytokines in the lung tissue of mice. Results are shown as means ± SD (*n* = 3). #*p* < 0.01 vs. no treatment (NT) group; ^∗^ *p <* 0.05 vs. LPS group; ^∗∗^ *p <* 0.01 vs. LPS group. Hor: hordenine; LPS: lipopolysaccharide; BALF: bronchoalveolar lavage fluid; IL-6: interleukin-6; IL-1β: interleukin-1β; TNF-α: tumor necrosis factor-α; MPO: myeloperoxidase; OD: optical density.

LPS significantly increased the protein secretion of IL-6, IL-1β, and TNF-α in the BALF and the mRNA expression levels of Il6, Il1β, Tnfa, iNOS, Cox2, and Mpo in the lung tissues. However, after Hor pre-treatment, the gene and protein levels of inflammatory mediators in the lung tissues were effectively suppressed, and Hor dose-dependently inhibited these inflammatory mediators. Based on these results, Hor alleviated ALI by inhibiting increases in inflammatory cytokine levels (Figure 3).

### Effect of Hor on RAW264.7 Cell Viability

**FIGURE 4:**
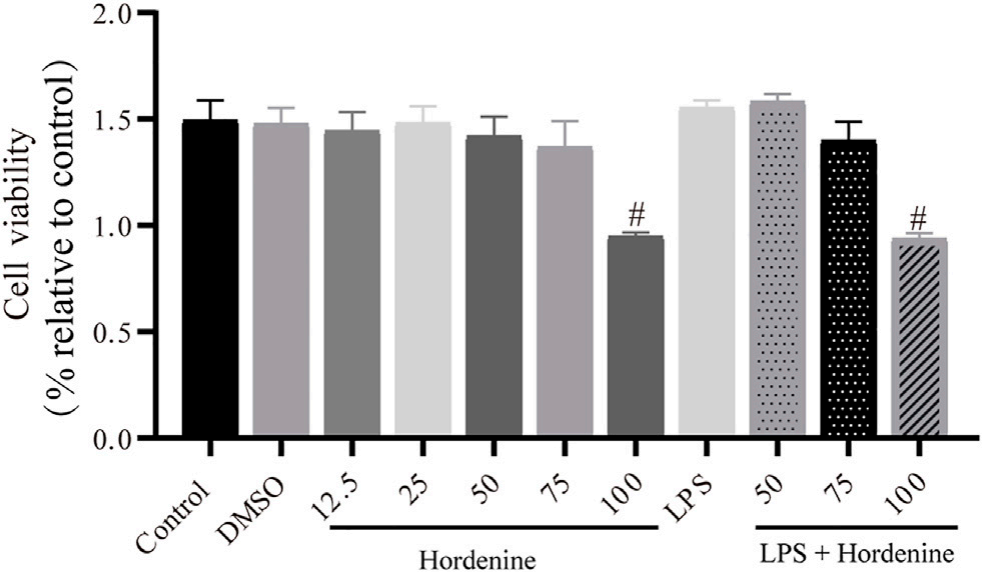
Effects of different concentrations of hordenine base with or without LPS on the viability of RAW264.7 cells. RAW264.7 cells were pre-treated with hordenine for 1 h and stimulated with LPS for 2 h. Cell viability was determined using the Cell Counting Kit-8 assay. Results are shown as means ± SD (*n* = 3). #*p* < 0.01 vs. control group. LPS: lipopolysaccharide; DMSO: dimethyl sulfoxide.

To further explore the effect of Hor on ALI, *in vitro* experiments were performed. Different concentrations of Hor (12.5, 25, 50, and 75 μg/ml) did not induce cytotoxicity. Additionally, LPS stimulation of the cells did not cause cytotoxicity. However, 100 μg/ml Hor stimulation induced cytotoxicity (Figure 4). Therefore, we used 50 and 75 μg/ml Hor for subsequent *in vitro* experiments.

### Hor Inhibited the Inflammatory Response in LPS-Induced RAW264.7 Cells

**FIGURE 5:**
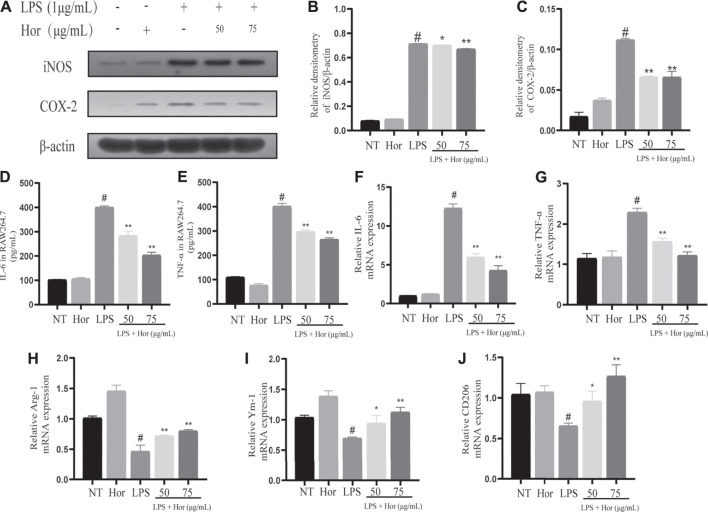
Hordenine inhibits LPS-induced inflammatory responses in RAW264.7 cells. RAW264.7 cells were incubated with Hor for 1 h before stimulation with LPS for 6 or 12 h. Radioimmunoprecipitation assay buffer or TRIzol was added to the cell samples to prepare protein or RNA samples. **(A–C)** Protein levels of COX-2 and iNOS in RAW264.7 cells detected by western blotting. **(D–E)** Levels of IL-6 and TNF-α protein in RAW264.7 cells. **(F–J)** The mRNA levels of M1 markers (IL-6 and TNF-α) and M2 markers (Arg-1, Ym-1, and CD206) in RAW264.7 cells. Results are shown as means ± SD (*n* = 3). #*p* < 0.01 vs. no treatment (NT) group; ^∗^ *p <* 0.05 vs. LPS group; ^∗∗^ *p* < 0.01 vs. LPS group. Arg-1: arginase one; CD206: mannose receptor; COX-2: cyclooxygenase-2; Hor: hordenine; IL-6: interleukin-6; iNOS: inducible nitric oxide synthase; LPS: lipopolysaccharide; TNF-α: tumor necrosis factor-α.; Ym-1: chitinase-3-like-3.

To further investigate the role of Hor in alleviating ALI, the expression levels of inflammatory mediators in LPS-induced RAW264.7 cells were examined. Significantly increased protein levels of iNOS and COX-2 were observed in LPS-treated RAW264.7 cells compared to those in the NT group (Figures 5A–C). LPS significantly induced the expression of IL-6 and TNF-α at the mRNA level as well as their expression and secretion at the protein level (Figures 5D–G). Furthermore, LPS inhibited the expression of M2 markers (Arg-1, Ym-1, and CD206) (Figures 5H–J). However, Hor pre-treatment effectively reduced the increased iNOS, COX-2, IL-6, and TNF-α levels and promoted the expression of Arg-1, Ym-1, and CD206. These results suggest that Hor alleviated ALI by inhibiting the inflammatory response.

### Hor Inhibited Activation of the AKT, NF-κB and MAPK Signal Pathways in LPS-Induced RAW264.7 Cells

**FIGURE 6:**
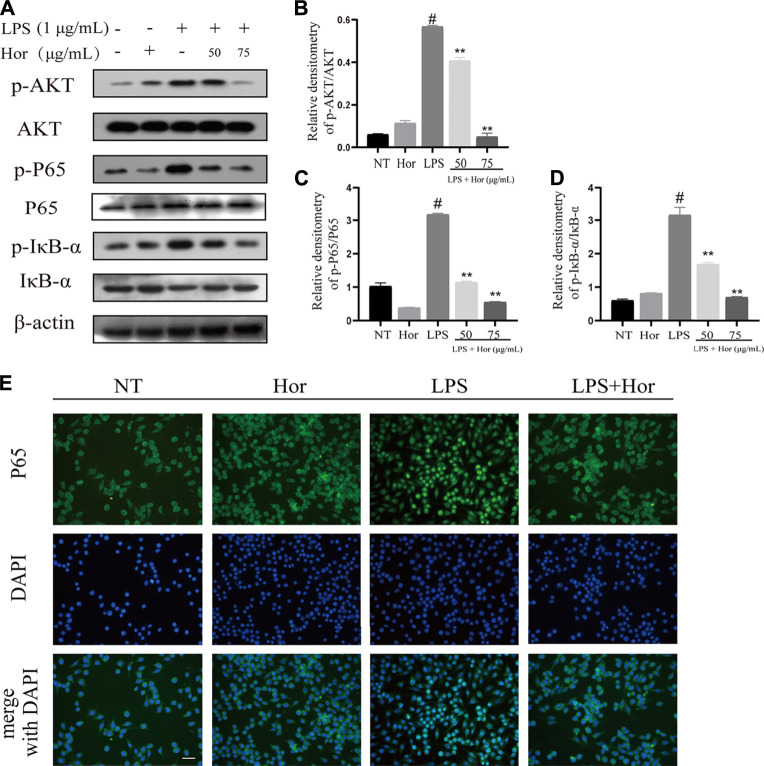
Hordenine inhibits phosphorylation of the AKT and NF-κB signaling pathways in LPS-induced RAW264.7 cells. Cells were pre-treated with hordenine for 1 h, followed by LPS treatment for 1 h, and cell proteins were collected. **(A)** Protein levels of p-AKT, p-p65, p-IκB, AKT, p65, and IκB in RAW264.7 cells. **(B–D)** Quantification of p-AKT, p-p65, and p-IκB by western blotting in RAW264.7 cells. **(E)** Translocation of p65 protein into the nucleus of RAW264.7 cells. Green represents p65 protein and blue represents the nucleus. Green fluorescence in the location of the nucleus was greater than that in the cytoplasm, indicating that p65 was translocated into the nucleus (magnification, × 400; scale bar = 100 μm). Results are shown as means ± SD (*n* = 3). #*p* < 0.01 vs. no treatment (NT) group; ^∗∗^ *p <* 0.01 vs. LPS group. DAPI, 4′,6-diamidino-2-phenylindole; Hor: hordenine; LPS: lipopolysaccharide; p-AKT: phosphorylated protein kinase B; p-IκB: phosphorylated inhibitor of nuclear factor-κβ; p-p65: phosphorylated p65.

**FIGURE 7:**
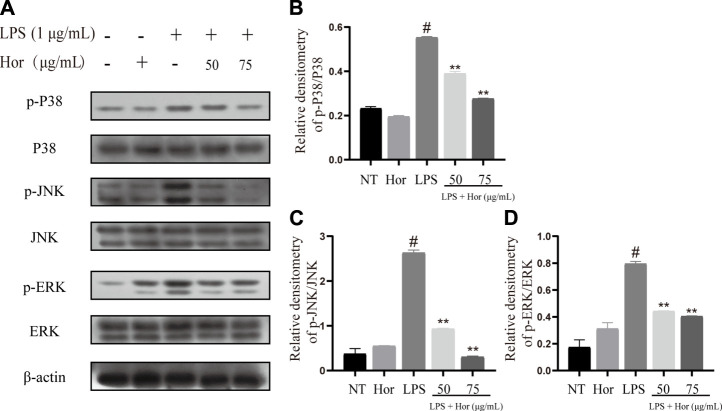
Hordenine inhibits activation of the MAPK signaling pathway in LPS-induced RAW264.7 cells. **(A)** Expression of p-p38, p38, p-JNK, JNK, p-ERK1/2, and ERK1/2 in RAW264.7 cells. **(B–D)** Relative density analysis of p-p38, p-JNK, and p-ERK1/2 protein bands in RAW264.7 cells; β-actin was used as an internal reference. Results are shown as means ± SD (*n* = 3). #*p* < 0.01 vs. no treatment (NT) group. ^∗∗^ *p <* 0.01 vs. LPS group. Hor: Hordenine; LPS: lipopolysaccharide; p-ERK1/2: phosphorylated extracellular signal-regulated kinase 1/2; p-JNK: phosphorylated c-Jun N-terminal kinase.

To explore the potential mechanism by which Hor alleviated ALI, various signaling pathways were examined. Compared to the NT group, the LPS treatment group showed significant induction of AKT, p65, and IκB protein phosphorylation in RAW264.7 cells (Figures 6A–D). Translocation of p65 protein into the nucleus was significantly induced by LPS (Figure 6E). However, Hor pre-treatment effectively alleviated increased phosphorylation levels of AKT, p65, and IκB, and translocation of p65 protein into the nucleus was significantly inhibited (Figure 6). Compared with those of the NT group, the LPS group significantly induced the phosphorylation of p38, ERK1/2, and JNK in RAW264.7 cells. However, Hor pre-treatment effectively alleviated the increased phosphorylation of p38, ERK1/2, and JNK. These results indicated that Hor alleviated ALI by inhibiting the phosphorylation of AKT, NF-κB, and MAPK (Figure 7).

### Molecular Docking Simulation of Hor With AKT

**FIGURE 8:**
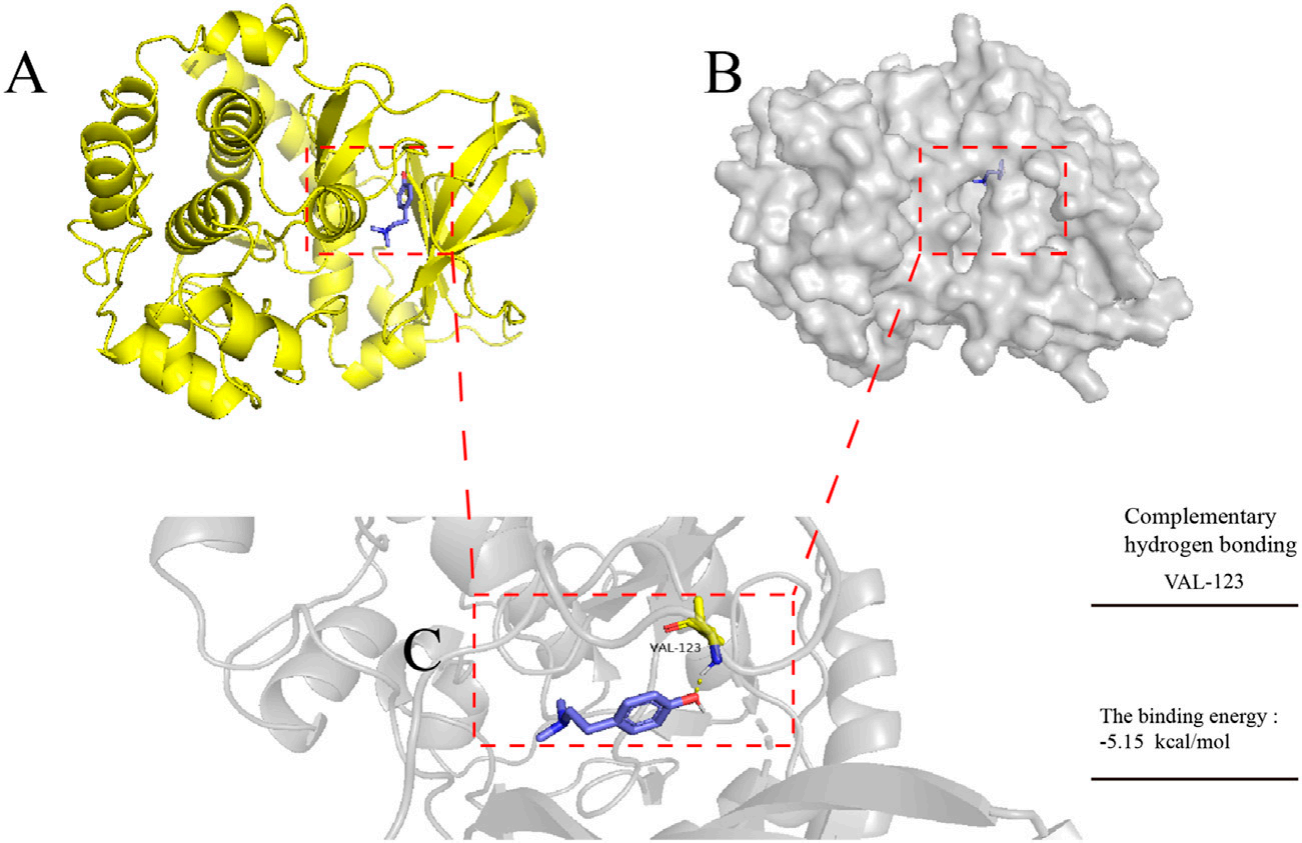
Molecular docking simulation of hordenine with AKT. AutoDock tools were used to predict the docking of protein kinase B (AKT) protein with hordenine, and PyMOL was used to plot the binding mode. **(A)** Three-dimensional (3D) model of AKT protein binding with a hordenine-cartoon pattern, AKT protein No. 3OW3. **(B)** Three-dimensional model of AKT protein binding to the hordenine-surface model. **(C)** Amino acid residues of AKT protein hydrogen bonding with hordenine. Blue represents hordenine and yellow represents the amino acid residues of AKT protein.

To explore the potential mechanism by which Hor alleviated ALI, the binding pattern and hydrogen bonds of Hor and AKT were predicted by molecular docking; the results revealed hydrogen bonding between Hor and AKT with a total energy of −5.15 kcal/mol, which was significant (Figure 8). These predictions suggested that Hor may alleviate ALI by inhibiting AKT phosphorylation.

## Discussion

In this study, an ALI model induced by LPS *in vivo* and *in vitro* was successfully constructed. The LPS-induced ALI model shows similarities to the pathological process of human ALI (Lv et al., 2017; Hou et al., 2018); therefore, in this study, LPS-induced *in vitro* and *in vivo* models were used to explore the effect and potential mechanism of Hor on ALI. We demonstrated that Hor effectively alleviated ALI, inhibited increased inflammatory mediators, and inhibited activation of inflammatory signaling pathways.

Lung tissue edema leads to enhancement of tissue inflammation and then destroys the normal physiological structure of the tissue, thus affecting normal physiological function (Komiya et al., 2017). Some studies showed that alleviating pulmonary hydrops is an effective approach for alleviating ALI (Hou et al., 2018; Huang et al., 2019). The current study showed that Hor alleviated LPS-induced lung edema and lung tissue damage. To further explore how Hor exerted these effects, inflammatory mediators were evaluated.

Inflammatory mediators are the main effectors of inflammation (Medzhitov, 2008; Lv et al., 2017). Inflammatory cytokines recruit large numbers of inflammatory cells to injured tissue and then induce a series of pathological processes, such as an inflammatory cytokine storm and respiratory burst, which damage the normal structure and physiological function of the tissue and aggravate the severity of the disease (Medzhitov, 2008; Kan et al., 2019). MPO is the main marker of neutrophil aggregation; inhibiting increased MPO levels effectively alleviates the inflammatory response (Aratani, 2018; Kan et al., 2019). iNOS is the key rate-limiting enzyme in NO synthesis; as an important signal regulatory molecule, it is closely related to the occurrence and development of diseases. Inhibiting increases in iNOS levels is useful for treating ALI (Zheng et al., 2019). COX-2 is a polyunsaturated fatty acid metabolic enzyme with a key role in the production of prostaglandins and is closely related to the occurrence and development of inflammation (Hu, 2003). Under normal physiological conditions, COX-2 expression is ordinarily very low, but it is significantly increased during inflammation, which promotes the synthesis of prostaglandins and induces cell damage (Hashemi Goradel et al., 2019). Inhibiting increased COX-2 levels can be used to treat ALI (Butt et al., 2016; Park et al., 2018a). In addition, the inflammatory cytokines IL-6, IL-1β, and TNF-α activate the inflammatory NF-κB signaling pathway, promote the secretion of inflammatory mediators, and expand the inflammatory response (Jimi et al., 2019). It follows that inhibiting the expression and secretion of IL-6, IL-1β, and TNF-α is a suitable approach for alleviating ALI. Our results showed that Hor inhibited the LPS-induced increase in inflammatory mediators in RAW264.7 cells, lung tissue, and BALF, and may have great potential for alleviating ALI. In addition, our study found that pre-treated Hor can reduce the polarization-related markers of M1 macrophages, while also enhancing the expression of M2 macrophage markers and promoting the polarization of macrophages to the M2 phenotype, which provides new evidence for further exploration of the anti-inflammatory mechanism of Hor.

To further explore the potential mechanism by which Hor alleviates ALI, the activation of inflammatory signaling pathways was examined. AKT/NF-κB is a classic signaling pathway (Manning and Toker, 2017; Ye et al., 2017). AKT plays an important role in the regulation of cell metabolism, growth, proliferation, survival, transcription, and protein synthesis, and is an important molecule in proinflammatory signaling (Manning and Toker, 2017). Studies have shown that AKT is a key molecule in the inflammatory response. External stimuli such as LPS can promote the phosphorylation of AKT. After phosphorylation of AKT, inflammatory signals are transmitted downstream, prompting the expression of several inflammatory cytokines, thus exacerbating the inflammatory response (Wang et al., 2017; Kan et al., 2019). Therefore, inhibition of AKT phosphorylation is a potential therapeutic strategy for alleviating inflammatory diseases. Many studies have provided evidence to support this point; for example, inhibition of AKT phosphorylation effectively alleviates inflammatory mastitis (Kan et al., 2019), inflammatory neurological disease (Huang et al., 2018), and inflammatory bowel disease (Chen et al., 2018). It has also been suggested that inhibition of AKT phosphorylation may be a potential strategy for relieving ALI (Hou et al., 2018). However, whether Hor can alleviate ALI by inhibiting the phosphorylation of AKT has not been reported. Through a molecular docking prediction, we found that there was an interacting hydrogen bond between Hor and AKT, which may inhibit the phosphorylation of AKT. Notably, our results show that Hor can inhibit LPS-induced phosphorylation of AKT, thus it may be a potential AKT inhibitor; however, this requires further investigation. In addition, AKT is an upstream factor of NF-κB, which further phosphorylates NF-κB signaling after activation. Thereafter, IκB protein is ubiquitinated and degraded, promoting p65 to enter the nucleus and directly interact with the promoter, resulting in transcription and translation of inflammation-related genes to maintain a high level of inflammation (Zhong et al., 2013; Meng et al., 2018; Pan et al., 2020). Our results showed that Hor effectively inhibited the LPS-induced activation of AKT and NF-κB, suggesting that Hor functioned via a mechanism involving AKT and NF-κB. Effective inhibition of AKT and NF-κB signal activation alleviates ALI (Pan et al., 2020). However, whether Hor can inhibit the NF-κB pathway by inhibiting AKT activation needs to be further confirmed.

**FIGURE 9:**
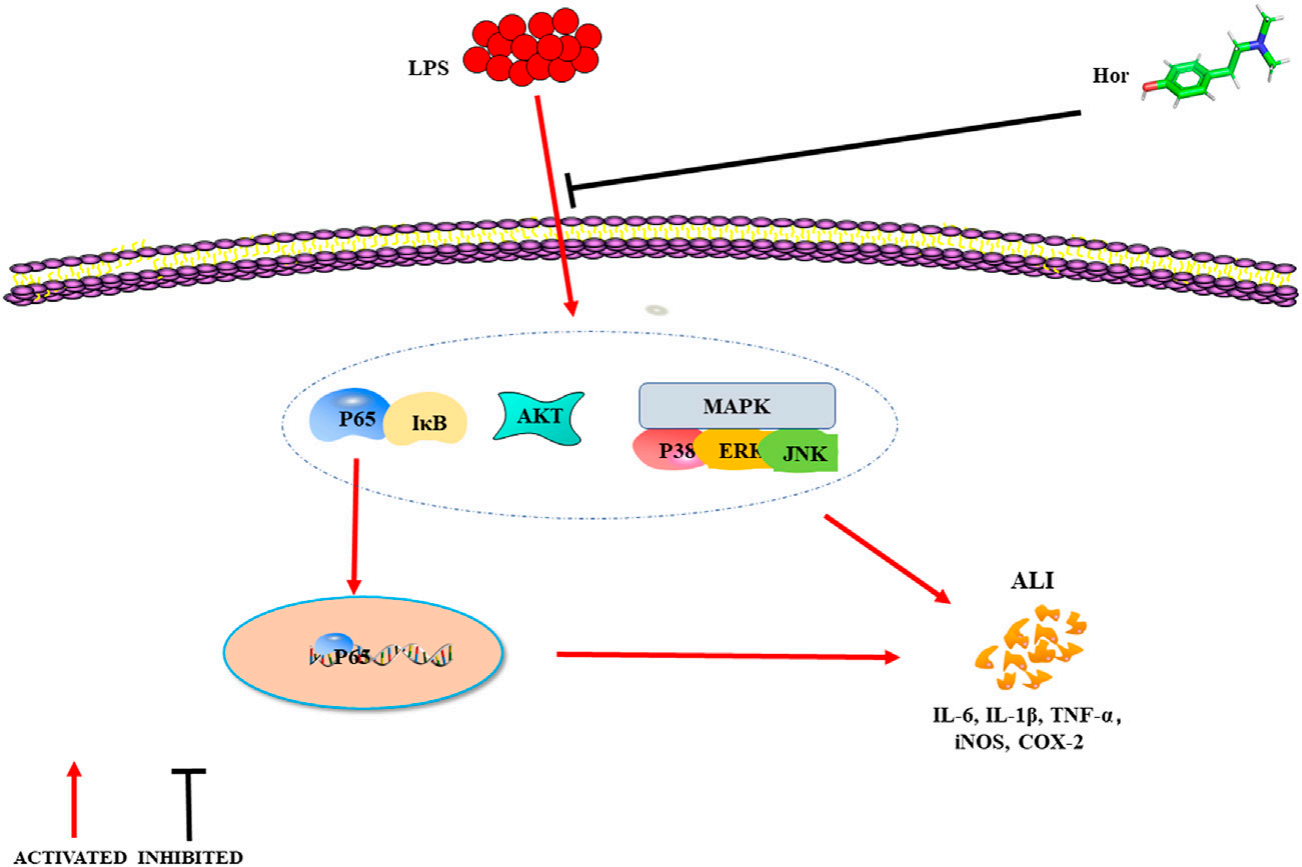
Mechanism of hordenine alleviating LPS-induced ALI. LPS successfully induces ALI in mice, and lung tissue injury was evident when the levels of inflammatory mediators increased. Hordenine alleviated LPS-induced ALI, inhibited the expression and secretion of inflammatory mediators, and relieved pulmonary tissue edema. The potential mechanism by which hordenine alleviates lung injury may be inhibition of AKT, NF-κB, and MAPK signaling activation. ALI: acute lung injury; LPS: lipopolysaccharide; AKT: protein kinase B; IκB: inhibitor of nuclear factor-κB; MAPK: mitogen activated protein kinase; ERK: extracellular signal-regulated kinase; JNK: c-Jun N-terminal kinase; IL-6: interleukin-6; TNF-α: tumor necrosis factor-α; iNOS: inducible nitric oxide synthase; COX-2: cyclooxygenase-2.

The activation of MAPK signaling was evaluated to investigate the potential mechanisms responsible for the effects of Hor on ALI. MAPK cascade activation is involved in multiple signaling pathways, mainly composed of p38, JNK, and ERK1/2 signals (Zhong et al., 2013). MAPK is an important transmitter of signals from the cell surface to the nucleus, where activation of MAPK signaling has a strong proinflammatory function (Fang and Richardson, 2005; Zhong et al., 2013). Phosphorylation of the p38 protein aggravates pneumonia in mice (Xing et al., 2019). ERK1/2 increases the adhesion of inflammatory cells and promotes the aggregation of inflammatory cytokines, thus aggravating the inflammatory response (Zhang et al., 2015; Park et al., 2018b). Furthermore, inhibition of JNK phosphorylation effectively alleviates ALI in mice (Zhang et al., 2015). These results show that Hor inhibited the phosphorylation of p38, JNK, and ERK1/2, indicating that inhibition of MAPK signaling was a potential therapeutic mechanism by which Hor alleviates ALI (Figure 9).

## Conclusion

Hor is a natural phytochemical extracted from germinated barley. It is currently challenging to establish accurate models of clinical disease. We established *in vitro* and *in vivo* models of ALI and clarified the mechanism and pathways of Hor in protecting against LPS-induced ALI, demonstrating its potential clinical application value. Hor effectively alleviated LPS-induced ALI. The potential mechanism involved the control of inflammatory mediator levels by inhibiting activation of AKT, NF-κB and MAPK signals.

## Data Availability

The original contributions presented in the study are included in the article/Supplementary Material, further inquiries can be directed to the corresponding authors.
