# Supplementary figures and images for: Hordenine Protects Against Lipopolysaccharide-Induced Acute Lung Injury by Inhibiting Inflammation
Source: Front Pharmacol. 2021 Sep 1;12:712232. doi: 10.3389/fphar.2021.712232 (PMC8440820; doi:10.3389/fphar.2021.712232)

**Figure 5**

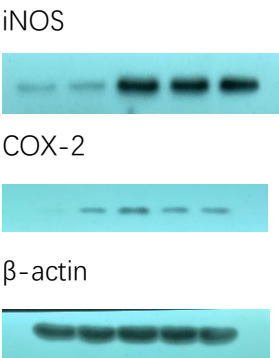

**Figure 6**

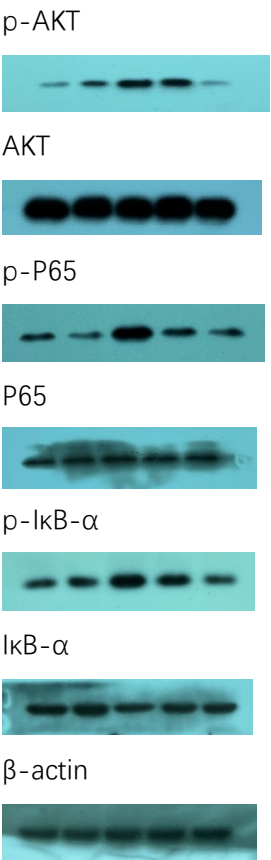

**Figure 7**

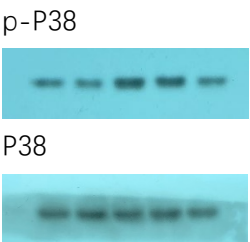

p-JNK

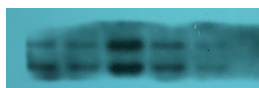

JNK

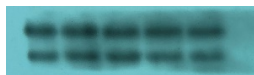

p-ERK

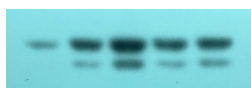

ERK

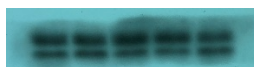

$\beta$ -actin

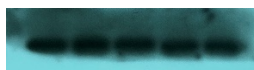

Supplement: Supplementary file 1 [file DataSheet1.PDF]
